# Synthesis of Magnetic Molecularly Imprinted Polymer Sorbents for Isolation of Parabens from Breast Milk

**DOI:** 10.3390/ma13194328

**Published:** 2020-09-29

**Authors:** Martyna Pajewska-Szmyt, Ewelina Biniewska, Bogusław Buszewski, Renata Gadzała-Kopciuch

**Affiliations:** 1 Department of Environmental Chemistry and Bioanalytics, Faculty of Chemistry, Nicolaus Copernicus University, 7 Gagarin St, 87100 Toruń, Poland; martynapajewska@interia.pl (M.P.-S.); e.ww.79@wp.pl (E.B.); bbusz@umk.pl (B.B.); 2 Interdisciplinary Centre of Modern Technologies, Nicolaus Copernicus University in Toruń, 4 Wileńska St, 87100 Toruń, Poland

**Keywords:** magnetic sorbents, molecularly imprinted polymers, parabens, breast milk, sample preparation, separation field

## Abstract

Magnetic molecularly imprinted polymers (MMIPs) are an invaluable asset in the development of many methods in analytical chemistry, particularly sample preparation. Novel adsorbents based on MMIPs are characterized by high selectivity towards a specific analyte due to the presence of a specific cavity on their polymer surface, enabling the lock–key model interactions to occur. In addition, the magnetic core provides superparamagnetic properties that allow rapid separation of the sorbent from the sample solution. Such a combination of imprinted polymers with a magnetic core has an innovative influence on the development of separation techniques. Hence, the present study describes the synthesis of MMIPs with 17β-estradiol used as a template molecule in the production of imprinted polymers. The as-prepared sorbent was used for a sorption/desorption study of five parabens from breast milk samples. The obtained results were characterized by sorption efficiency exceeding 92%, which shows the high affinity of the analytes to the functional groups on the sorbent. The final determination of the selected analytes was done with high-performance liquid chromatography using a fluorometric detector. The determined linearity ranges for selected parabens were characterized by high determination coefficients (r^2^ from 0.9992 to 0.9999), and the calculated limit of detection (LOD) and limit of quantification (LOQ) for the identified compounds were low (LOD from 1.1–2.7 ng mL^−1^; LOQ from 3.6–8.1 ng mL^−1^), which makes their quantitative analysis in real samples feasible.

## 1. Introduction

To improve sample preparation processes and, consequently, the entire analytical procedure, there is a need to synthesize new sorbents. In recent years, in the world of nanoparticles, molecularly imprinted polymers (MIP) have been synthesized as new innovative sorbents. The development of sorbents and the imprinted particle allows proposing innovative solutions in separation and purification [1,2,3,4]. MIPs are characterized as artificial bio-receptors or as antibody–antigen systems due to the mechanism based on the selective binding of specific molecules in a special cavity, which is similar to the lock–key model [5]. Although a number of methods to synthesize such analytical tools have been proposed so far, the general scheme of operation is similar. At the beginning, polymer covalently or non-covalently bound with a template molecule is prepared, and then the template molecule is removed, which leads to leaving a specific recognition cavity. These sites are made by functional groups, which create a complementary shape so that a bond can be made with the target analyte [5,6,7]. Therefore, the following reagents are needed for the synthesis: functional monomer (necessary to create a pre-polymerization complex), crosslinker, and a template. Although such sorbents already significantly affect the quality of separation, it is possible to modify them further by adding magnetic properties to receive magnetic molecularly imprinted polymers (MMIP). In this case, a magnetic core must be prepared, and a typical material used here is magnetite (Fe_3_O_4_). The magnetic particles for the core are distinguished by a large surface area-to-volume ratio, and their mean size is around 10 nm. The core is then covered with a specific polymer layer that ensures selectivity towards the target analyte [8]. First of all, sorbents with magnetic properties allow easy separation of the sorbent from the sample solution when an external magnetic field is used [9]. As a consequence, the use of this type of sorbent in a sample preparation method eliminates such steps as centrifugation or filtration, which consequently reduces the run time of the analytical procedure. Due to the facilitated separation of the sorbent from the sample solution and the fact that it does not need to be packaged in a solid-phase extraction column, the as-prepared MMIPs are used for dispersive solid-phase extraction. The dispersion of sorbents in the sample significantly affects the extraction efficiency due to better access of the analyte to recognition cavities [10,11,12].

Parabens are esters of p-hydroxybenzoic acid with different alkyl groups, including methyl-(MeP), ethyl-(EtP), propyl-(PpP), and butyl-(BuP) paraben. Chain length affects their physicochemical properties and increases their antimicrobial activity [13,14,15]. Since they exhibit antimicrobial and antifungal properties, these synthetic chemicals have been widely used as additives in personal care products, such as shampoos, bath gels or body creams, and others [16,17]. Unfortunately, research also indicates that they may have toxic effects on the human body, causing, e.g., changes in the hormonal system or skin allergies [16,18]. Since parabens are still commonly used as preservatives, and considering their long-term side-effects, there is a need to monitor these compounds in the environment and, in particular, in biological samples, one of which can be human milk. Because breastfeeding is the main source of nutrition for infants, and during this period, children are particularly vulnerable, lactating women can expose children to hazardous paraben levels [19]. There have already been studies focusing on this issue reporting the use of different methods of sample preparation for human milk with traditional solvents and sorbents, such as liquid–liquid extraction (LLE) [20], solid-phase extraction (SPE) [21], ultrasound-assisted extraction (UAE) [22], dispersive liquid–liquid microextraction (DLLME) [23] as well as quick easy cheap effective rugged safe (QuEChERS) technique [24,25]. Only a few reports on using molecularly imprinted polymers in different matrices [26,27,28,29,30,31] have appeared so far. Thus a topic of great scientific interest is to synthesize a new sorbent based on magnetic nanoparticles modified by a layer of molecularly imprinted polymers and to apply such sorbent to breast milk samples. It should be mentioned that molecularly imprinted polymers are still an innovative approach to sample preparation, and it is still a challenge for analytical chemistry to apply them to real samples with complex matrix composition. It can be observed that in standard solutions, the efficiency of sorption or desorption is very high. Moreover, concentrations of analyte used in experiments are often much higher than those expected in real samples. However, when a real sample, e.g., an environmental or a biological one, is taken for testing, many other components from the group of fats or proteins can be found there as well. Thus, in this case, the behavior of a sorbent with a specific cavity may differ significantly from what has been observed for a standard solution. Therefore, research directly related to the human milk matrix can significantly expand our knowledge related to sample preparation.

Therefore, the main goal of this research was to prepare and characterize novel magnetic molecularly imprinted polymers for the separation of five parabens form breast milk. To the best of our knowledge, the present study is the first attempt to use 17β-estradiol (E2) as a template for MIPs dedicated to paraben isolation. This molecule was selected as a template because the study’s aim was to extract five parabens varying in length of the attached alkyl chain. Moreover, the use of a different template than the analyte allows reducing the risk of the so-called leaching of residue template and can also provide multiple specific cavities, which can affect the adsorption capacity.

Methacrylic acid (MAA) was used as the functional monomer, ethylene glycol dimethacrylate (EGDMA) as the crosslinking agent, and azobis(isobutyronitryl) (AIBN) as the initiator to a synthesis procedure of ultrasound-assisted polymerization. The final determination of selected analytes was carried out with high-performance liquid chromatography using a fluorescence detector (HPLC-FLD).

## 2. Materials and Methods

### 2.1. Reagents and Materials

Iron(III) chloride hexahydrate (FeCl_3_·6H_2_O) (97%), iron(II) chloride tetrahydrate (FeCl_2_·4H_2_O) (98%), 17β-estradiol (98%), oleic acid (90%), methacrylic acid (MAA), and ethylene glycol dimethacrylate (EGDMA) were purchased from Sigma–Aldrich (Sigma–Aldrich, Steinheim, Germany); azobis (isobutyronitryl) (AIBN) from Fluka (Sigma–Aldrich, Steinheim, Germany); sodium dodecyl sulfate (SDS) from POCh (Avantor Performance Materials Poland S.A., Gliwice, Poland; glacial acetic acid from J.T. Baker (Mallinckrodt Baker Inc., Phillipsburg, NJ, USA); ammonia solution 25% from POCh, Poland; acetone, and methanol from POCh (Avantor Performance Materials Poland S.A., Gliwice, Poland); acetonitrile from Merck (Merck KGaA, Darmstadt, Germany). All analytical standards of parabens—benzylparaben (99%), butylparaben (99%), methylparaben (98%), propylparaben (99%), ethylparaben (99%), were purchased from Sigma–Aldrich. A basic solution was prepared from the standard weights at specific concentrations for individual analytes: MeP—1028 μg mL^−1^; EtP—998 μg mL^−1^; PpP—1330 μg mL^−1^; BuP—1008 μg mL^−1^, BnP—1010 μg mL^−1^. Standard solutions necessary to determine the calibration curves were prepared by diluting the stock solution.

Human milk donated as surpluses from Human Milk Bank was used as a real matrix for test application of obtained sorbent. The real sample was received from the Human Milk Bank, Ludwik Rydygier Provincial Polyclinical Hospital in Toruń. All subjects gave their informed consent for inclusion before they participated in the study. The study was conducted in accordance with the Declaration of Helsinki, and the protocol was approved by the Bioethics Committee (Nicolaus Copernicus University in Toruń functioning at Collegium Medicum in Bydgoszcz) of KB 351/2019.

### 2.2. Instrumentation

Fourier transform infrared (FT–IR) spectra were recorded using the FT-IR Vertex 70 V spectrophotometer with Hyperion 1000/2000 microscope (Bruker Optik, Ettlingen, Germany). These measurements were performed in the frequency interval of 4000–400 cm^−1^ with a resolution of 1.4 cm^−1^. The X-ray diffractometer (XRD) patterns were recorded using an XRD analyzer (Philips X”Pert with X’Celerator Scientific detector, Malvern Panalytical Ltd., Malvern, UK). The diffractogram was obtained by using Cu-Kα radiation in the range 10° < 2θ° < 80° with steps of 0.02° and an acquisition time of 1.0 s/step. The porosity parameters of polymers (S_BET_—specific surface area; V_p_—pore volume; S_p_—pore size) were determined by the low-temperature nitrogen adsorption-desorption method at 77 K using a Model 1800 Sorptomatic instrument (Carlo Erba, Milan, Italy). Transmission electron microscopy (TEM) and scanning electron microscopy (SEM) images were obtained on a Tecnai F20 X-Twin (FEI Company, Hillsboro, OR, USA) transmission electron microscope and model 1430 VP (LEO Electron Microscopy Ltd., Thornwood, NY, USA) scanning electron microscope, respectively. The SEM samples were prepared by dropping a dilute suspension of powder on a glass. The surface was coated with a thin gold film under vacuum before microscopy scanning. In the case of TEM analysis, samples of sorbents were deposited on copper grids (400 mesh) coated with carbon. An ultrasonic bath, USC 1200T (VWR, Leuven, Belgium), was used for sample preparation.

Liquid chromatographic analyses of parabens were performed using a high-performance liquid chromatography system (Model 1100, Agilent Technologies Deutschland GmbH, Waldbronn, Germany) equipped with DAD (Model 1100) and fluorescence detector (FLD) (Model 1260) (Agilent Technologies Deutschland GmbH, Waldbronn, Germany). OpenLab CDS software (Agilent, Santa Clara, CA, USA) was used for data acquisition and quantification. The ACE 3 C18-AR column (Advanced Chromatography Technologies Ltd, Aberdeen, Scotland) (Advanced Chromatography Technologies, 150mm × 4.6 mm; d_c_ = 3 μm) was used as the stationary phase for separation. Chromatographic separation was performed by gradient elution with (A) acetonitrile and (B) water: 0–0.2 min 40% A; 0.2–10 min 41% A; 10–12 min 100% A. Before the next injection of the sample, the column was washed with 100% of the organic solvent (acetonitrile) over 8 min and then conditioned (17 min). The flow rate was 1 mL min^−1^, and the injection volume was 10 μL. The fluorescence detector (FLD) photomultiplier tube (PMT) gain was set to 16 with analytical wavelengths as λ_ex_ = 254 nm and λ_em_ = 310 nm. The chromatographic method was developed for this particular application and has been validated.

### 2.3. Validation of Analytical Procedure

According to the International Conference of Harmonization (ICH) guidelines on validation of analytical procedures (ICH, 2005) [32], selected validation parameters were determined, such as linearity, limit of detection (LOD), limit of quantification (LOQ), precision, reproducibility, specify and selectivity. The calibration curves were developed to determine the linearity of the chromatographic method. For this purpose, standard solutions of the investigated parabens were prepared at nine concentration levels, and each concentration was injected six times. Then the response standard deviation (δ) of the curve and the slope of the calibration curve (S) were used to calculate the limits of detection (LOD) and quantification (LOQ). The standard deviation of the response was calculated from the standard deviation of the intercept y of the regression line. The precision of the method was determined with reference to the areas under the peak (A), and the repeatability—with reference to the retention times (t_R_) of the particular parabens. Both parameters were expressed by standard deviation (SD). Standard deviation was also used to evaluate the error of sorption and desorption studies.

### 2.4. Synthesis Procedure of Magnetic Molecularly Imprinted Polymer

#### 2.4.1. Synthesis of Magnetic Nanoparticles

Eight point seven micromolar FeCl_2_·4H_2_O and 15.7 mmol FeCl_3_·6H_2_O were placed in a round-bottom flask and dissolved in 80 mL of double deionized water (DDW). Then the solution was degassed with a stream of nitrogen (N_2_). After that, the content was heated to 80 °C in an oil bath under the reflux, and 10 mL of ammonia solution was added drop by drop. Then the mixture was stirred at 80 °C for 30 min. After this time and cooling the content of the flask, the particles were separated by an external magnet and washed with water to pH = 7, then with methanol, and finally, with acetone. The obtained magnetic nanoparticles (MNPs) were dried in a vacuum drying oven (BINDER VDL 53, BINDER GmbH, Tuttlingen, Germany) at 60 °C for 12 h.

#### 2.4.2. Modification of the Magnetic Core

After the synthesis of magnetic particles, the next step was to modify them. The Fe_3_O_4_@OA@SDS was obtained with the following procedure: 0.017 mmol of Fe_3_O_4_ was dispersed in 40 mL DDW, and the solution was treated with ultrasounds at 60 °C for 5 min with the maximum power of the ultrasonic device set to 9. Next, 0.86 mmol of oleic acid (OA) was divided into three parts, and after each portion (92 L) was added, the solution was sonicated. Finally, the solution of SDS (0.93 mmol) in DDW (40 mL) was added to the content, and again, the mixture was sonicated at room temperature for 10 min with the power set to 9.

#### 2.4.3. Preparation of Magnetic Molecularly Imprinted Polymers

**Figure 1:**
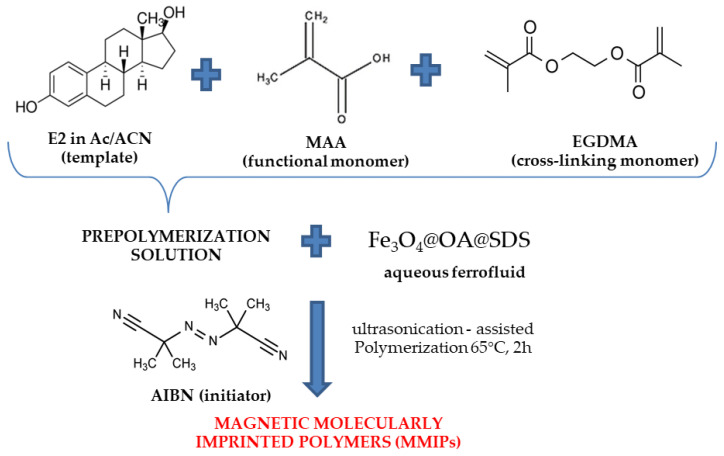
Scheme of synthesis of magnetic molecularly imprinted polymers.

The synthesis of magnetic polymers with the imprinted molecule was performed by ultrasound-assisted polymerization (Figure 1). The modified magnetic core (Fe_3_O_4_@OA@SDS) was used for this purpose. 17β-estradiol (0.5 mmol) was used as a template molecule and 4 mmol methacrylic acid (MAA) as a functional monomer. The template molecule was dissolved in the mixture of acetone:acetonitrile (1:3), followed by the addition of the functional monomer. The solution was degassed by nitrogen stream and stirred for 2 h in the dark.

After this time, 20.0 mmol of EGDMA was added as a crosslinking monomer. The resulting pre-polymerization solution (20 mL) was combined with previously obtained ferrofluid (20 mL). The solution was placed in an ultrasound bath for 1 min. The next step was to add the initiator, such as AIBN (0.5 mmol) and degassed the mixture with nitrogen stream. The whole polymerization mixture was sonicated for 2 h at 65 °C, with power set to 9. The obtained magnetic molecularly imprinted polymers were collected by an external magnetic field—a neodymium magnet. For this purpose, the magnet was placed on the outside of the container with the magnetic sorbent. The template molecule was removed with the mixture of methanol:acetic acid (9:1), then washed with water until pH = 7, and finally, with methanol and acetone. The magnetic imprinted polymer (MMIP) was dried under vacuum at 60 °C for 12 h. Magnetic non-imprinted polymer (MNIP) was prepared according to the same procedure, without adding a template molecule.

### 2.5. Sorption and Desorption Studies of Parabens

#### 2.5.1. Binding Analysis of MMIP

The study on the sorption of parabens with the obtained polymers used a sample of 500 L of milk. One hundred liters of the standard mixture at 30–60 ng mL^−1^ specific parabens and 400 L of DDW were added to the milk sample. The contents were shaken for 10 min. To eliminate proteins contained in the milk, the samples were frozen for 30 min and then centrifuged. The solution was transferred to a clean Eppendorf tube with 3 mg of sorbent. The contents were placed in an ultrasonic bath for 10 min to facilitate the sorption process and then shaken for 5 min. Each experiment was conducted in three repetitions. Then, with the use of an external magnet, the solution was collected for HPLC analysis.

#### 2.5.2. Selection of the Amount of Sorbent

To investigate the sorption of parabens on different amounts of sorbent (MMIP or MNIP), 500 μL of mother’s milk was used as a model matrix. One hundred microliters of the standard mixture was added to the sample at a concentration of 30–60 ng mL^−1^ for individual parabens, followed by 400 μL of DDW. The Eppendorf tube contents were shaken for 10 min. To eliminate proteins contained in milk, the samples were frozen for 30 min and then centrifuged. The solution was transferred to a clean Eppendorf tube with the appropriate amount of sorbent (1 to 4 mg). The contents were placed in an ultrasonic bath for 10 min to facilitate the sorption process and then shaken for 5 min. Each experiment was conducted in three repetitions. The solution was then collected with an external magnet for HPLC analysis.

#### 2.5.3. Sorption

To investigate the sorption of parabens on MMIPs, 500 μL of breast milk was used as the model matrix, and 100 μL of the standard mixture at concentrations of 30–60 ng mL^−1^ for particular parabens was added to the milk. The contaminated milk was diluted with 400 μL DDW, and the content was shaken for 10 min. As the model matrix was human milk, the elimination of proteins from the sample should also be taken into account. For this purpose, the sample content was frozen for 30 min; then, the sample was centrifuged. The solution was moved to another Eppendorf tube and then applied to 3 mg of sorbent. The contents were placed in an ultrasonic bath for 10 min to improve the sorption process, followed by shaking for 5 min. Then, using an external magnet, the solution was collected for HPLC analysis.

#### 2.5.4. Desorption

Parabens adsorbed on the sorbent were desorbed using a mixture of methanol:acetic acid (9:1 *v*/*v*). For this purpose, 100 μL of desorption mixture was applied to the sorbent remaining after sorption. The content was placed in an ultrasonic bath for 10 min, followed by shaking for 5 min. Finally, an external magnet was used to separate the extract from the sorbent. The extract was transferred to an Eppendorf tube and placed in a vacuum centrifuge (Labconco™ CentriVap DNA Vacuum Concentrator, Labconco Corporation, Kansas City, MO, USA). The content was evaporated to dryness for 2 h. Then the residue was dissolved in 200 μL of acetonitrile (ACN).

## 3. Results and Discussion

### 3.1. Effectiveness of the Performed Syntheses

To demonstrate the success of the syntheses, its effectiveness was calculated for individual stages. For this purpose, the weight method was used for determining the efficiency of sorbent synthesis. The efficiency is expressed as a percentage of the results (weight) of the materials obtained divided by the theoretical weight resulting from the number of reagents used, and the results are presented in Table 1.

### 3.2. Analytical Performance Characteristics

**Figure 2:**
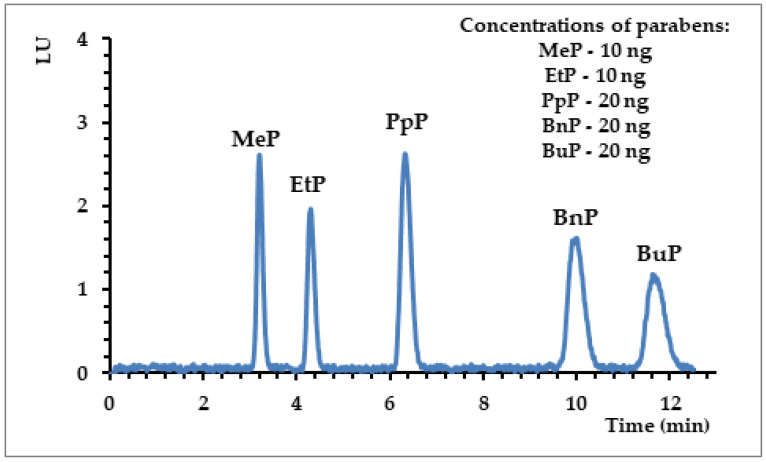
Selected high-performance liquid chromatography using a fluorescence detector (HPLC-FLD) chromatograms obtained for parabens. Conditions of analysis: ACE 3 C18-AR column (150 × 4.6 mm; d_c_ = 3 μm); gradient elution with (A) acetonitrile and (B) water: 0–0.2 min 40% A; 0.2–10 min 41% A; 10–12 min 100% A; flow rate: 1 mL min^−1^; FLD: λ_ex_ = 254 nm/λ_em_ = 310 nm.

The results of HPLC-FLD (detailed conditions of analysis described in Section 2.2) analyses were validated and are presented in Table 2. The determined ranges of linearity for the particular analytes indicated high values of the correlation coefficient (r^2^ > 0.9997), which confirmed that the applied technique (HPLC-FLD) maintained linearity within the tested concentration range. Moreover, the method was characterized by a low value of standard deviations for peak area precision (< 8%) and for the repeatability of retention times (1.2%), which indicates that the chromatographic conditions were stable. Considering that the detection and quantification limit is an essential parameter to determine the sensitivity of the method, the obtained limits were at low levels (LOD < 3 ng mL^−1^ and LOQ < 8 ng mL^−1^), which made it possible to analyze parabens at very low concentration levels with the use of the developed HPLC-FLD method (Figure 2).

### 3.3. Physicochemical Characteristics of As-Prepared MMIPs

All stages of the research were monitored and verified with the use of instrumental techniques such as porosimetry, X-ray diffraction, scanning electron microscope (SEM), transmission electron microscope (TEM), and infrared spectroscopy (IR).

The textural properties were studied by nitrogen adsorption–desorption measurements at 77 K. Low-temperature adsorption–desorption of nitrogen made it possible to measure the amount of the adsorbed gas on the surface of the polymer with defined mass, depending on the pressure of the said gas. The specific surface area (m^2^ g^−1^) and pore volume (cm^3^ g^−1^) were calculated by means of the Barrett, Joyner and Halenda (BJH) method. The specific surface area (S_BET_), pore volume (V_p_), and pore size (S_p_) of MMIPs (S_BET_ = 45.8 m^2^ g^−1^; V_p_ = 0.31 cm^3^ g^−1^; S_p_ range from 20 to 350 nm) are larger than those of MNIPs (S_BET_ = 38.2 m^2^ g^−1^; V_p_ = 0.28 cm^3^ g^−1^ S_p_ range from 15 to 300 nm), which is most likely due to the existence of imprinted cavities in MMIPs. It can be seen that the surface area values are not large for both the imprinted and the non-imprinted polymers.

**Figure 3:**
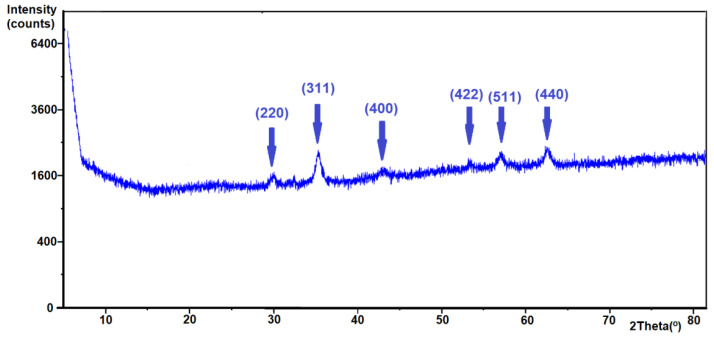
X-ray diffractograms of the obtained Fe_3_O_4_.

The X-ray diffraction pattern of the nanoparticles is presented in Figure 3. A series of characteristic peaks with higher intensity for MNPs was obtained with the described synthesis method, which was connected to an inverted spinel structure of Fe_3_O_4_ [33,34]. The pattern of Fe_3_O_4_ had six characteristic peaks at 2θ = 30.2, 35.7, 43.1, 53.5, 57.0, and 62.7 corresponding to the (220), (311), (400), (422), (511), and (440) crystal faces, respectively.

**Figure 4:**
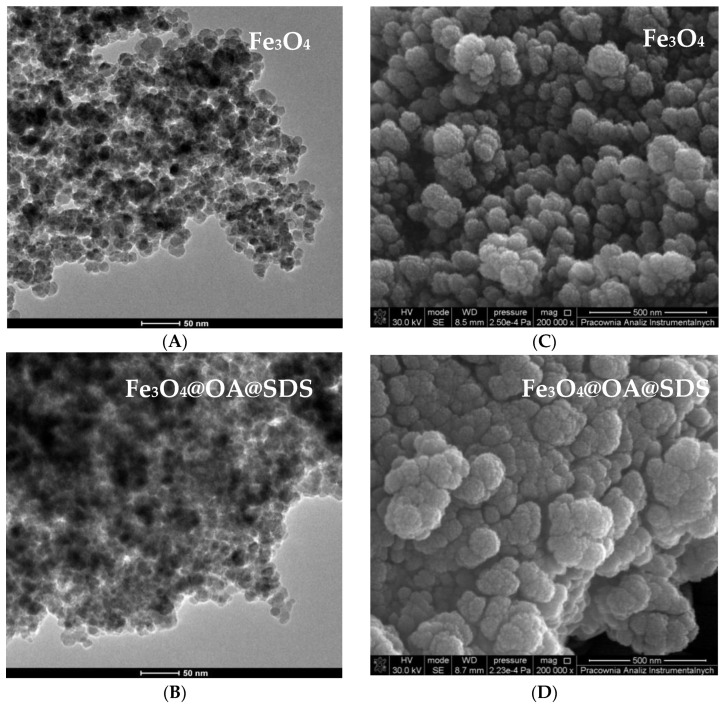
Transmission electron microscopy (TEM) (**A**,**B**) and scanning electron microscopy (SEM) (**C**,**D**) image of Fe_3_O_4_ and Fe_3_O_4_@OA@SDS.

The surface morphology of MNPs Fe_3_O_4_@SDS@OA, MMIP, and MNIP was investigated with SEM and TEM. The TEM images (A and B) are presented in Figure 4. The uncoated Fe_3_O_4_ (A) were aggregated, which was expected due to the large surface-to-volume ratio and, consequently, the high surface energy of nanoparticles [33,34,35]. In Figure 4B, a coating of Fe_3_O_4_ by oleic acid can be observed. A large zone of dark area may be observed, which can be associated with greater particle distribution. Images from scanning electron microscope (Figure 4C,D) show the size difference between uncoated MNPs and those with an OA layer. The increase in the size of magnetic particles is related to their oleic acid surrounding.

**Figure 5:**
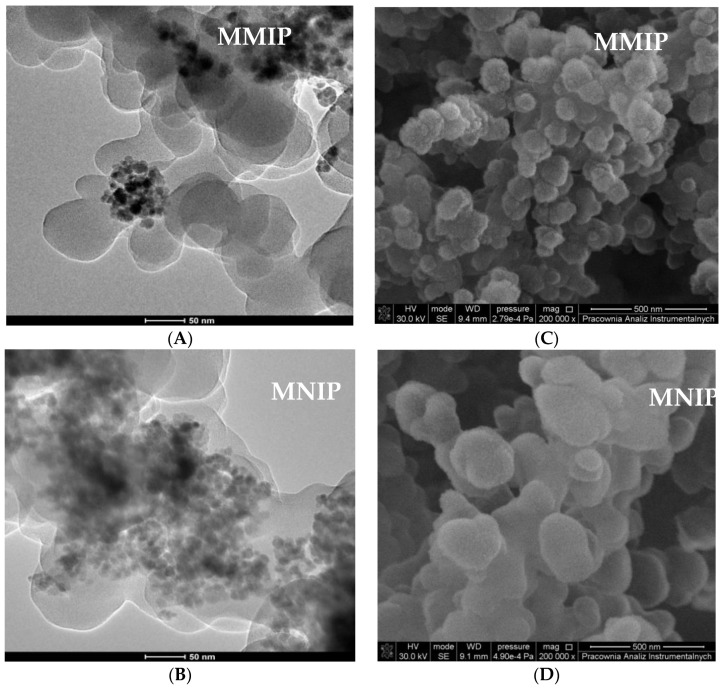
TEM (**A**,**B**) and SEM (**C**,**D**) image of magnetic molecularly imprinted polymers (MMIP) and magnetic non-imprinted polymer (MNIP).

Figure 5 shows the particles after the next modification process, i.e., polymerization aiming to obtain a molecularly imprinted polymer layer on a magnetic core. According to the TEM images (Figure 5A,B), after another modification in which the layer of polymer was immobilized on a magnetic particle, it had a more homogeneous distribution than basic Fe_3_O_4_ and Fe_3_O_4_@SDS@OA [36]. Meanwhile, after the removal of the template molecule, MMIPs were characterized by a more visible porous structure with cavities [37,38]. Moreover, when comparing MMIP and MNIP, one can see that the former were smaller, exactly due to the fact that the template was washed out (Figure 5C,D).

**Figure 6:**
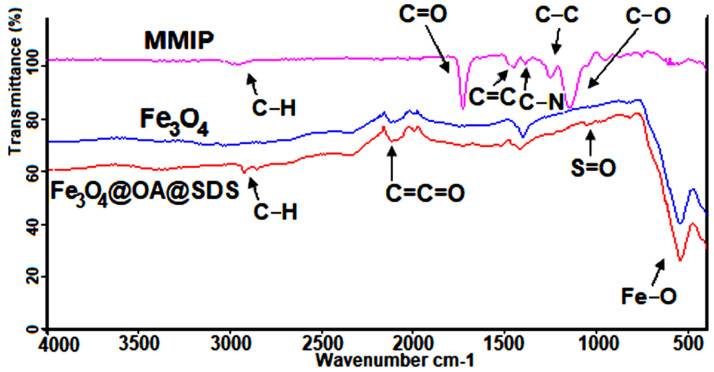
Fourier transform infrared (FT-IR) spectrum of Fe_3_O_4_ (**blue**), Fe_3_O_4_@OA@SDS (**red**), and MMIP (**pink**).

The FT-IR results (Figure 6) confirmed the efficiency of the synthesis of magnetic molecularly imprinted polymers through the major steps of modification. First of all, attention should be paid to the characteristic absorption band at approximately 550 cm^−1^, which originates from vibrations of Fe–O. After modifying the magnetic core with OA, IR of as-prepared nanoparticles presented a weak band of C–H vibration in the area between 2900 and 3050 cm^−1^. Finally, MMIPs were characterized by an absorption band at 1050–1200 cm^−1^, which belonged to the C–O group that originated from the crosslinker EGDMA used in their production. These results confirmed the success of the polymerization process. At 1600–1700 cm^−1^ a carboxylic bond (C=O) of MAA can be observed. Moreover, the IR of MMIP spectrum also contained bands for C–N at 1300–1150 cm^−1^, for C=C at 1350–1550 cm^−1^, for C=C=O stretching vibration at 2150 cm^−1^, and for C–H stretching vibration at 2900–3050 cm^−1^ [36,37].

### 3.4. Optimization of MNIP and MMIP Parameters Influencing Paraben Adsorption

#### 3.4.1. The Analysis of Binding in MNIP and MMIP

The amount of polymer-bound parabens was calculated from its concentration in the MMIP samples compared with the MNIP samples, where Q_MMIP_ and Q_MNIP_ are the amounts of the bound parabens on the polymer with and without the printed template, respectively. The amount of parabens adsorbed on MMIPs (or MNIPs) was calculated according to the following equation:

(1) $$Q=\frac{{(C}_{0}-C_{F})\cdot V}{m}$$

where *C*_0_ (ng mL^−1^) and *C_F_* (ng mL^−1^) are the initial and final concentrations of the paraben solution, respectively; *V* (mL) is the volume of the paraben solution, and *m* (mg) is the mass of the polymer.

**Figure 7:**
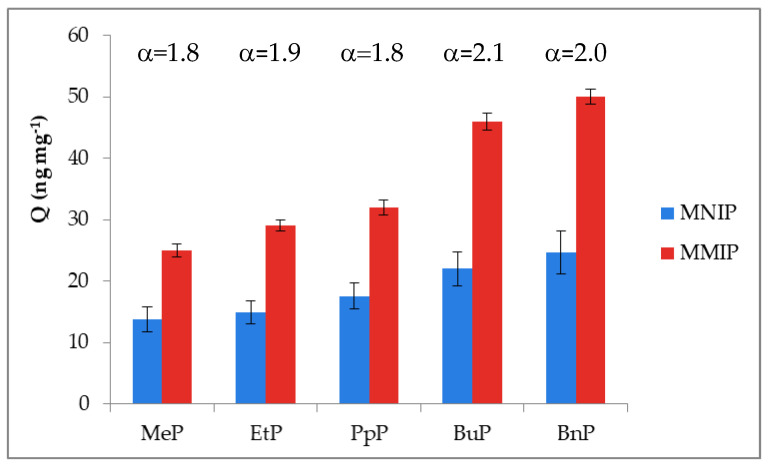
Imprinting effect of MMIPs as demonstrated by experiments on binding parabens, where: *α* = *Q_MMIP_/Q_MNIP_).* Adsorption conditions: *V* = 0.6 mL, *m_MMIP_* or *m_MNIP_* = 3 mg, *c_parabens_* = 30–60 ng mL^−1^, in milk sample.

The ligand-binding efficiency (*α*) was calculated as the percentage of compounds bound by MMIP to the percentage of analytes bound by MNIP. The results summarized in Figure 7 confirmed that MIPs had higher adsorption capacity as compared to the five parabens adsorption by MNIP. MNIP only adsorbed parabens on the surface due to the lack of complementary cavities in the polymer network. The complementary cavities created in the MMIP were capable of distinguishing target molecules based on their size, shape, and functional group distribution.

#### 3.4.2. The Sorption Levels and the Amount of MNIP and MMIP

**Figure 8:**
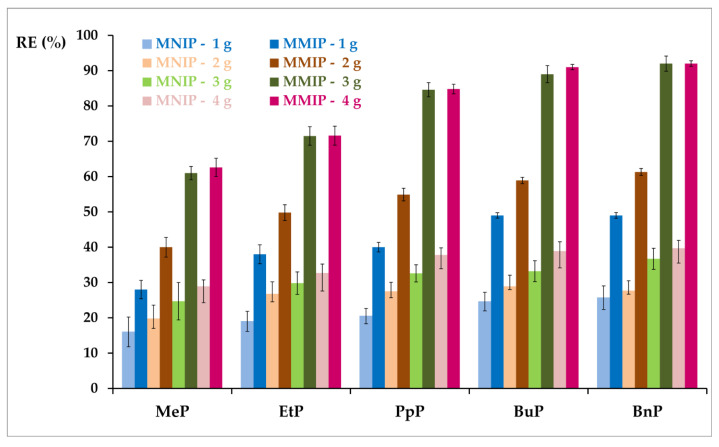
The percentage of five paraben removal efficiency (RE) at different amounts of sorbents (MMIP and MNIP) for the following adsorption conditions: initial concentration of each of the determined parabens: 30–60 ng mL^−1^; solution volume = 0.6 mL; t = 23 °C.

To determine the effect of the amount of sorbent on the sorption of selected parabens, weights of 1, 2, 3, and 4 mg MNIP or MMIP were used (the experimental conditions are described in Chapter 2.5.2). Figure 8 shows the dependence of the amount of sorbent on the value of the removal efficiency of selected parabens. The lowest removal values for all parabens were obtained for the batch quantity of 1 and 2 mg of the sorbent used (RE_MNIP-1g_ from 16 to 26%; RE_MNIP-2g_ from 20 to 28%). At higher levels (3 or 4 mg) of the sorbent, RE_MNIP_ BnP was approx. 40%, while for the other parabens were lower values (MeP, RE_MNIP_ for a maximum of 30%).

When 1 and 2 mg MMIP were used, RE values ranged from 28% to 61% of the MeP growing tendency to the BnP. A significant increase in RE was observed for the amounts of 3 and 4 mg, which ranged from 62% to 92%, with the same trend of increase from MeP to BnP. The difference in RE for 3 mg and 4 mg of MMIP was negligible, so further research used 3 mg of the adsorbent. Moreover, higher reproducibility was observed with the use of MMIP (SD did not exceed 5%), while for MNIP it ranged from 5% to 10%.

#### 3.4.3. Result of Sorption/Desorption Studies

**Figure 9:**
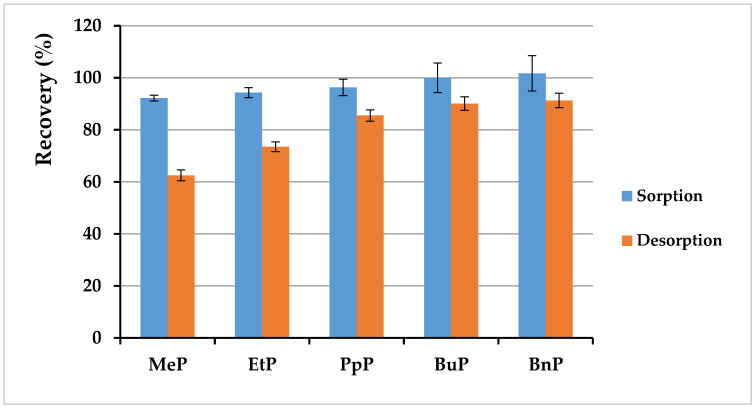
Results of sorption and desorption studies for particular parabens in human milk (each experiment conducted in three repetitions).

After the successful sorbent synthesis, the next step was to check the effectiveness of paraben sorption on the obtained MMIPs. The procedure was described in Section 2.5.3 and Section 2.5.4, and the experiment results are presented in Figure 9. It ought to be considered that this research was carried out with human milk as matrix; the milk sample was spiked with solutions of paraben standards and homogenized before the whole procedure was carried out, so the effectiveness of the obtained sorbent was determined with regard to both the matrix composition and subsequent steps included in sample preparation. As was described in Section 2.5.3 and Section 2.5.4, milk was diluted with water, which was related to using water as a loading solvent. This choice was associated with the requirement to obtain non-specific adsorption of the analyte via hydrophobic interactions. According to the obtained data, sorption was very effective at the levels of 92–102%, with a standard deviation lower than 7%. Obtaining a recovery above 100% for MMIP may suggest incomplete purification of the extract by sorption of structurally similar analytes and their elution at the same retention time. Moreover, a mixture of methanol:acetic acid (9:1 *v*/*v*) was used for desorption for two reasons. First of all, a solvent for elution should be selected in which the analytes dissolve well as in methanol. Furthermore, the addition of acetic acid to the elution mixture increases the chance of breaking hydrogen bonds between the analyte and functional groups in the selective cavity. As a result, the efficiency of desorption with CH_3_OH:CH_3_COOH (9:1) was lower than sorption. The applied mixture enabled obtaining recoveries in the range of 62–91% with a low standard deviation (1.9–2.8%). However, it should be noted that the repeatability was very satisfactory, which seems promising for further research.

Comparison of these studies with others found in the literature is not easy due to the fact that, first of all, only a few of them describe the synthesis and application of MMIPs for paraben separation. Second, there are even fewer reports on the application of this technology to breast milk samples. You et al. [26] used real sample fruit juices and obtained good recoveries of 81.9%. They coated magnetic nanoparticles with a representative of the silane group, and buthylparaben was used as a template molecule. One of the limited examples of the use of this type of sorbent for human milk testing is the research by Melo and Queiroz [27]. The researchers synthesized MIP with sol–gel polymerization, and used benzylparaben as the template with 3-aminopropyltrimetoxysilane as the functional monomer and tetraethyl orhtosilicate as the crosslinker. The accuracy of the obtained method for separation of methylparaben, ethylparaben, and propylparaben ranged from 86 to 117%. Propylparaben was used as a template molecule to synthesize MIPs, and also to isolate this compound by Vicario et al. [29]; they used bulk polymerization where MAA and EGDMA were used as functional monomer and crosslinker, respectively. The obtained recovery was 97% and was determined using industrial wastewater samples (Table 3). As can be seen from the results collected in Table 3, only one text was found, which described the use of a polymer with an imprinted particle with magnetic properties [26]. In most cases, sample preparation was based on the use of the obtained sorbent in the SPE [27,29,31,32,33,34,35,36,37,38,39,40,41,42,43], which requires packing an SPE cartridge. In our opinion, the use of the sorbent in bulk form as a dispersive solid-phase extraction allows a quicker sample preparation. In addition, the magnetic core makes it easier to separate the analyte from other components of the sample, without the need for centrifugation or filtration.

Most of the mentioned studies used one of the parabens as a template, while in our research, we decided to use a dummy template in the form of 17β-estradiol, which was a novel approach. To our knowledge, no such template has been used for sorbents dedicated to such xenoestrogens as parabens. This choice was motivated by the need to obtain a sorbent that would allow the sorption of all five parabens. Owing to the fact that particular parabens are characterized by different lengths of alkyl chain substituted to the benzene ring, 17β-estradiol was selected as it has analogous function groups as –CH_3_ or –OH. The use of another compound as a template for the sorption of a group of analytes significantly affects the extraction efficiency by enhancing the presence of active sites and also minimizes the risk of the template being incompletely washed out. In addition, the use of another compound as a template minimizes the risk of the “template leakage”. Compared to other studies, and taking into account that human milk is a matrix not used at all in the MIPs studies, the obtained results provide a very good basis for further consideration and continued work on similar aspects of MMIPs with a focus on human milk as the sample.

The use of new sorbents can mainly reduce the sample preparation time and the number of reagents used. Magnetic molecularly imprinted polymers can be an essential alternative to the traditional methods used for extracting parabens from milk. Liquid–liquid extraction and solid-phase extraction have often been used for this purpose. LLE requires the use of a large amount of sample, which in the case of breast milk, is not demanded, whereas in solid-phase extraction, traditional sorbents often do not allow complete purification of the extract, and the use of reagents is much higher than in dispersive solid-phase extraction [19,21,44,45]. In connection with the above, the use of a new sorbent, such as MMIP, with a selective cavity allows obtaining specific interactions, better cleaning of the sample (minimizing the matrix effect), and the possibility of skipping time-consuming centrifuging and filtration.

## 4. Conclusions

Synthesis of new sorbents is an extremely intriguing field, which is, above all, very necessary for the development of analytical chemistry. The adsorbent obtained in this research was based on magnetic molecularly imprinted polymers. The high level of sorption shows that with the use of 17β-estradiol as a template, a selective cavity that showed a high affinity for the five parabens was created on the sorbent surface. The use of milk as a matrix is also a valuable aspect of this work due to the complexity of the sample solution.

## Figures and Tables

**Table 1:** Determination of the effectiveness of the performed syntheses for magnetic core modified magnetic core, and molecularly imprinted polymers.

Nanoparticles	Effectiveness (%)
magnetic core	
Fe_3_O_4_^1^	35.44
modified magnetic core	
Fe_3_O_4_@OA@SDS ^2^	35.90
molecularly imprinted polymers	
MNIP ^3^	69.34
MMIP ^4^	72.83

^1^ Fe_3_O_4_—iron(II,III) oxide (magnetic core), ^2^ Fe_3_O_4_@OA@SDS—oleic acid (OA) and sodium dodecyl sulfate (SDS) functionalized Fe_3_O_4_, ^3^ MMIP—magnetic molecularly imprinted polymer, ^4^ MNIP—magnetic non-imprinted polymer.

**Table 2:** Validation parameters determined for the high-performance liquid chromatography using a fluorescence detector (HPLC-FLD) method.

Analyte	Linear Equation	Correlation Coefficient (r^2^)	Linear Range (ng mL^−1^)	LOD ^1^ (ng mL^−1^)	LOQ ^2^ (ng mL^−1^)
MeP ^3^	y = 0.1839x + 0.3800	0.9997	10.28–411.20	1.57	4.71
EtP ^4^	y = 0.1784x + 0.3869	0.9997	9.98–399.20	1.41	4.23
PpP ^5^	y = 0.1770x + 0.4086	0.9999	19.95–798.00	2.13	6.39
BuP ^6^	y = 0.1705x + 0.4900	0.9998	20.20–808.00	2.51	7.53
BnP ^7^	y = 0.1353x + 0.3913	0.9998	20.16–806.40	2.66	7.98

^1^ LOD—limit of detection, ^2^ LOQ—limit of quantification, ^3^ MeP—methylparaben, ^4^ EtP—ethylparaben, ^5^ PpP—propylparaben, ^6^ BuP—butylparaben; ^7^ BnP—benzylparaben.

**Table 3:** Example of the molecularly imprinted polymers for paraben extraction from various samples.

Abb. of Analyte	Functional Monomer	Crosslinking	Template	Matrix	MMIP ^1^ or MIP ^2^	R ^3^ (%)	Analytical Technique	Linear Range (ng mL^−1^)	LOD ^4^ (ng mL^−1^)	Ref.
MeP ^5^, EtP ^6^, PpP ^7^, BuP ^8^	AA ^9^	EGDMA ^10^	BuP	fruit juices	MMIP	73–89	LC-UV ^11^	100–1000	21–28	26
MeP, EtP, PpP	APTMS ^12^	TEOS ^13^	BnP ^14^	human milk	MIP	86–117	LC-UV	10–150	3–6.1	27
PpP	MAA ^15^	EGDMA	PpP	personal care products	MIP	97	LC-UV	8–500	2.4	29
MeP, EtP, iPrP ^16^, PpP, iBuP ^17^, BuP	MAA	DVB ^18^	BnP	solid environmental sample	MIP	80–90	LC-UV	0.5–25 ^19^	0.2–0.3 ^20^	31
BuP	4-VP ^21^	EGDMA	BuP	river water	MIP	>97	LC-MS ^22^	n.i. ^23^	n.i.	39
MeP, EtP, PpP, BuP	4-VP	EGDMA	BnP	plasma	MIP	n.i.	LC-MS/MS ^24^	1–50	0.1–0.2	40
2-HB ^25^	APTES ^26^	TEOS	2-HB	cosmetics	MIP	87–105	LC-UV	n.i.	n.i.	41
BA, EtP, MeP, PpP	MAA	EGDMA	EtP	soy	MIP	88–111	LC-UV	500–10,000	16–35	42
p-HB ^27^	AA	EGDMA	PA ^28^	plant material *(Melissa officinalis)*	MIP	16–82	LC-DAD ^29^	n.i.	n.i.	43
MeP, EtP, PpP, BuP, BnP	MAA	EGDMA	17β-estradiol	human milk	MMIP	92–102	LC-FLD ^30^	10–808	1.1–2.7	This study

^1^ MMIP—magnetic molecularly imprinted polymer; ^2^ MIP—molecularly imprinted polymer; ^3^ R—recovery; ^4^ LOD—limit of detection; ^5^ MeP—methylparaben; ^6^ EtP—ethylparaben; ^7^ PpP—propylparaben; ^8^ BuP—butylparaben; ^9^ AA—acrylamide; ^10^ EGDMA—ethylene glycol dimethacrylate; ^11^ LC-UV—liquid chromatography with ultraviolet detector; ^12^ APTMS—3-aminopropyltrimethoxysilane; ^13^ TEOS—tetraethyl orthosilicate; ^14^ BnP—benzylparaben; ^15^ methacrylic acid; ^16^ iPrP—isopropylparaben; ^17^ iBuP—isobutylparaben, ^18^ DVB—divinylbenzene; ^19^ linear range (ng g^−1^); ^20^ detection limits (ng g^−1^); ^21^ 4-VP—4-vinylpyridine; ^22^ LC-MS—liquid chromatography-mass spectrometry; ^23^ n.i.—not indicated; ^24^ LC-MS/MS—liquid chromatography with tandem-mass spectrometry; ^25^ 2-HB—2-hydroxybenzoic acid; ^26^ 3-aminopropyltriethoxysilane; ^27^ p-HB—p-hydroxybenzoic acid; ^28^ PA—protocatechuic acid; ^29^ LC-DAD—liquid chromatography with diode-array detector; ^30^ LC-FLD—liquid chromatography with fluorescence detector.
